# Soluble form of carbonic anhydrase IX (CA IX) in the serum and urine of renal carcinoma patients

**DOI:** 10.1038/sj.bjc.6601264

**Published:** 2003-09-09

**Authors:** J Závada, Z Závadová, M Zat'ovičová, L Hyršl, I Kawaciuk

**Affiliations:** 1 Institute of Molecular Genetics, Academy of Sciences of the Czech Republic, Flemingovo nám. 2, 16637 Prague, Czech Republic; 2 Institute of Virology, Slovak Academy of Sciences, Dúbravská cesta 9, 84245 Bratislava, Slovakia; 3 Clinic of Urology, Charles University, 2nd School of Medicine, 150 00 Prague, Czech Republic

**Keywords:** carbonic anhydrase IX, tumour antigens, cancer diagnostics

## Abstract

Tumour-associated protein carbonic anhydrase IX (CA IX) has two major forms. One is a cell-associated, transmembrane protein seen on Western blots as a twin band of 54/58 kDa, expressed in gastric mucosa and in several types of cancer. The other is a soluble protein s-CA IX of 50/54 kDa, which is released into the culture medium or into the body fluids, most likely by proteolytic cleavage of the extracellular part from transmembrane and intracellular sequences. While TC media of CA IX-positive tumour cell lines or short-term cultures of tumour explants contain a relatively high concentration of s-CA IX (20–50 ng ml^−1^), the level of this antigen in blood serum and urine of renal clear cell carcinoma patients is about 1000 × lower. The concentration of CA IX in the blood and in urine varies within wide limits and there is no obvious correlation with tumour size. After nephrectomy, s-CA IX is cleared from the blood within a few days. Only an extremely low concentration of CA IX was detectable in the sera and in urine of control individuals.

Carbonic anhydrase IX (CA IX) protein (previously named as MN or MN/CA IX) is a cell membrane protein (Pastoreková *et al*, 1992; Závada *et al*, 1993), which is ectopically expressed in various human tumours, mostly carcinomas, for example, cervical, renal, colorectal, lung, mammary and others (Liao *et al*, 1994, 1997; Saarnio *et al*, 1998). It has been suggested that CA IX may serve as a biomarker in early stages of tumorigenesis; now it is turning out to be induced in certain tumours by hypoxia, which is connected with poor prognosis (Chia *et al*, 2001; Giatromanolaki *et al*, 2001; Koukorakis *et al*, 2001). In renal clear cell carcinoma (RCC), synthesis of CA IX is switched on by the loss of suppressor gene VHL (Ivanov *et al*, 1998). Until recently, the identification of CA IX in formaldehyde-fixed, paraffin-embedded tumour sections has been based on immunohistochemical analysis using MAb M75.

On the other hand, there were no reports on detecting CA IX in body fluids of cancer patients. For the assays of soluble antigens, which are usually present only in extremely low concentrations, two antibodies are needed, preferably monoclonal, directed to different epitopes. One of them serves for concentrating the antigen and the other for its detection and quantitation. However, all efforts to raise antibodies specific for epitopes different from the M75 epitope have failed. This represented the crucial problem, which we succeeded to solve.

Our approach was based on the following consideration: sequencing of *Car9*, the mouse homologue of human *CA9* gene, revealed that the N-terminal, proteoglycan-like (PG) domain of the protein shows almost no homology between man and mouse. The carbonic anhydrase (CA) domain is relatively conserved in evolution. Therefore, the mouse will recognise human PG as ‘non-self’ and be able to respond to it immunologically, but it will not produce antibodies reacting with human CA domain. However, mice with the disrupted *Car9* gene (knockout) should be able to produce antibodies against different antigenic sites of the CA IX protein.

Indeed, Zat'ovičová *et al* (2003), taking advantage of *Car9*−/− mice constructed by Ortová-Gut *et al* (2002), was able to produce a series of potent monoclonal antibodies (mAbs) specific for different antigenic sites, many of which are located in the CA domain (e.g. the V-10 antibody used in this report). The epitope of our original M75 antibody is located in the PG domain of CA IX and its amino-acid sequence is PGEEDLP (Závada *et al*, 2000).

Using newly obtained mAbs, we were able to develop tests for the s-CA IX antigen. To start with, we decided to use tumour cell cultures as an easy model system. The first set of questions we started to ask were: (1) Do the tumour cells containing cell-associated CA IX also shed this antigen into the medium during cultivation? (2) Is the soluble form of CA IX different from cell-associated molecules? (3) What is the concentration of CA IX in the media? (4) Do short-term cultures of human tumours also shed s-CA IX, and if they do, how much of it? (5) Can soluble CA IX be detected in the serum and urine of the patients? (6) How fast is the clearance of CA IX from the blood and from urine after surgical removal of the tumour? (7) What is the dependence of CA IX concentration in body fluids on the tumour size? (8) Do the blood and urine of healthy individiuals also contain CA IX?

In the present experiments, we chose the patients with RCC because these tumours usually express CA IX to a high level and in a high proportion of the patients.

## MATERIALS AND METHODS

### Patients and controls

The study included 50 patients (28 men and 22 women, mean age 63. 2 years, range 36–79) with newly diagnosed RCC. All patients routinely underwent ultrasound and CT before surgery to obtain information for the standard staging protocol. Serum and urine from these patients were obtained before surgery and 5 days after it. In some patients, a sample of tumour tissue after nephrectomy was used for short-term RCC cultures. In all patients the diagnosis was confirmed histologically. In total, 18 patients served as controls; these had been admitted to the hospital for other urological conditions than RCC (infections, urinary stones, other urological tumours, etc.). Other control sera were obtained from healthy blood donors. The study had been approved by the Departmental Ethics Committee.

### Cell and tumour cultures and media

The cell line HT29 (DSMZ ACC299) derived from colorectal carcinoma and A498 (ATCC No. HTB-44) derived from RCC were grown in Dulbecco's modified Eagle's medium (DMEM) supplied with 10% foetal calf serum (FCS, Gibco). Short-term cultures of the tumours were fresh tumour excisions cut in 1 mm pieces, rinsed with phosphate-buffered saline (PBS, pH 7.2), suspended in DMEM with 10% FCS (about 50–100 mg of fresh weight of tumour fragments in 5 ml of the medium) and incubated in 5 cm Petri dishes at 37°C in a 5% CO_2_ incubator. Cell lysis buffer constitutes PBS with 1% Igepal CA-630 (Sigma), 0.25% deoxycholate and proteinase inhibitors (1 mM phenylmethylsulphonyl fluoride and 200 trypsin-inhibiting units of Trasylol per ml).

### Immunological reagents and methods

#### Monoclonal antibodies

M75 specific for the PG region of CA IX has been produced by Pastoreková *et al* (1992) and V-10 specific for CA domain, by Zat'ovičová *et al* (2003). The isotype of mAb M75 is IgG 2b and that of V-10 is IgG 2a. The IgG was purified from hybridoma TC fluid by affinity chromatography as previously (Závada *et al*, 2000). For the concentration of immune complexes formed by the mAbs with CA IX, antigen from TC media or cell extracts Protein A-Sepharose (Sigma) was used: the mAb-CA IX complexes from human sera or urine were adsorbed to anti-mouse IgG-Agarose (Sigma). The immunoperoxidase conjugate M75-Px was prepared using the peroxidase labelling kit (Roche Diagnostics, GmBH, Mannheim, Germany).

Immunoprecipitation of s-CA IX from human sera and urine was carried out as follows: to 0.75 ml of serum or 10 ml of urine (both clarified by centrifugation) were added 2 *μ*g of M75 IgG and allowed to react for 60 min at room temperature. Following this, each sample was supplied with 50 *μ*l of 50% pre-washed anti-mouse IgG agarose beads and the suspension was agitated in a rotator for 4 h or overnight at +4°C. Then the beads with bound mouse IgG and immune complexes were centrifuged (1200 r.p.m.2 min^−1^), the pellet was resuspended in 1 ml PBS with 0.05% Tween 20 (PBS-T, Sigma) centrifuged as above and the beads were heated at 100°C for 5 min in 60 *μ*l of Laemmli sample buffer supplied with 1.5–2% of FCS (for reasons explained in the last paragraph of Results).

#### ELISA

The ‘sandwich’ was composed of these layers: (1) Microtitre plates or strips were coated with mAb V-10 IgG, 5 *μ*g ml^−1^ in 50 mM carbonate buffer pH 9.6 which was adsorbed 3 h at room temperature, (2) test antigen dilutions in PBS with 1% FCS were adsorbed overnight at +4°C, (3) M75-Px conjugate 1 : 5000 in PBS with 1% FCS was allowed to bind for 2 h at room temperature, (4) orthophenylene diamine (OPD, Sigma), 1 mg ml^−1^ in 100 mM phosphate/citrate buffer pH 5.0+1 *μ*l ml^−1^ of 30% H_2_O_2_ (Sigma), was allowed to react 30 min in the dark at room temperature. After steps 1–3, the plates were washed × 4 with PBS-T. The reaction was terminated by adding two drops of 2 N H_2_SO_4_ per well. We have used the MaxiSorp strips or plates (NUNC, Denmark).

#### Western blot analysis

Sodium dodecyl sulphate – polyacrylamide gel electrophoresis and Western blotting were performed as described before (Závada *et al*, 2000). Carbonic anhydrase IX antigen on the blots was visualised by M75-Px conjugate, followed by enhanced chemiluminescence (ECL) and exposure to X-ray film Medix XG (FOMA, Czech Republic), with maximum sensitivity to yellow-green light.

## RESULTS

### Cell-associated and soluble CA IX in cell cultures

**Figure 1:**
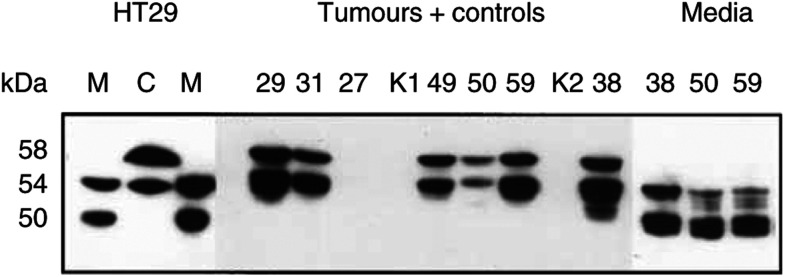
Western blot analysis of CA IX in culture media or in extracts from cells, tumours and control tissues. HT29: M=medium, C=cell extract. Numbers above the remaining lanes=codes for the patients; K1, K2=control kidneys; no. 27=angiolipoma; all other lanes=RCC. The lanes were loaded with 25 *μ*l of media or with 12.5 *μ*l of cell or tumour extracts adjusted to 1 mg of total protein per ml.

**Figure 2:**
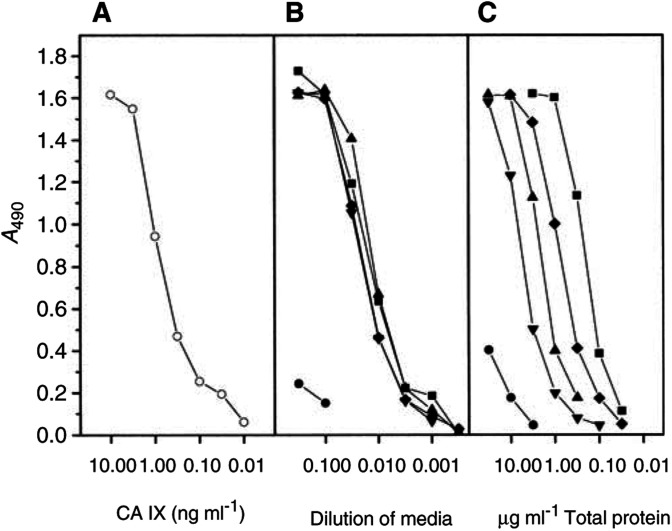
ELISA test for CA IX antigen. The numbers of tumour materials correspond to the patients in Figure 1. HT29 cells grew in a 5 cm Petri dish, supplied with 5 ml of media, exchanged 2 days before harvest. The extract from the cell monolayer in this culture contained 5 mg of total protein. Extracts from the tumours were prepared directly from the excisions, without previous cultivation *in vitro*. Media from the cultures of tumour fragments were harvested after 2 days. (**A**) Purified CA IX protein (described in Závada *et al*, 2000); (**B**) TC media; (**C**) extracts from cells and tumours; ○=purified CA IX; •=A498 cells; ▪=HT29 cells; patients no. ▴=38; ▾=50; ⧫=59.

Various human tumour cell lines, when grown to a high density, produce CA IX seen on Western blots as a ‘twin’ protein p54/58 (Pastoreková *et al*, 1992; Závada *et al*, 1993). The present results are consistent with these reports (Figure 1

). The HT29 cell line derived from colorectal carcinoma shows 54 and 58 kDa bands of CA IX on Western blots. Media from these cell cultures also contain CA IX, but the molecules of the soluble form are somewhat shorter, with two bands of 50 and 54 kDa. The total content of CA IX in the cell extract is about 10x higher than in the medium, as determined by ELISA (Figure 2

). The A498 cells are poor producers of CA IX.

### CA IX in tumour extracts and in media of short-term cultures of RCC

Western blots of the extracts from RCC tumours resemble blots of tumour cell cultures. They also contain two bands of 54 and 58 kDa and the proportion of CA IX per total protein is somewhat lower than in HT29 cells (Figures 1 and 2). All the six RCC tumour extracts were strongly CA IX positive. Control kidneys and a non-RCC tumour, an angiolipoma, did not contain any CA IX.

Shedding of s-CA IX was examined in short-term cultures: the medium from a suspension culture of tumour fragments was harvested after 2 days and analysed by ELISA and Western blotting. Again, both the concentration and *M*_r_ of s-CA IX were comparable with the cultures of permanent tumour cell lines such as HT29.

### CA IX in the serum of RCC patients

There were no problems with detection and quantitation of the CA IX protein in extracts from cells or tumours and in culture media. The concentration of CA IX was sufficient for direct analysis by Western blotting or by ELISA. Control cell extracts or media showed no false positivity or nonspecific bands.

However, it was necessary to concentrate the CA IX protein from the sera and urine of RCC patients before gel electrophoresis and Western blotting.

We tested 30 samples of sera from RCC patients, 14 sera of patients with tumours other than RCC, six sera of nontumour patients suffering from various urological diseases and 42 sera of healthy blood donors. Sera evaluated as positive were found mainly in the RCC group. Out of total 30 RCC sera, 12 scored as ‘positive’ (40%). The RCC group included one sarcomatous RCC, which was CA IX positive. Among 14 non-RCC tumour patients, one was positive (papillary carcinoma).

**Figure 3:**
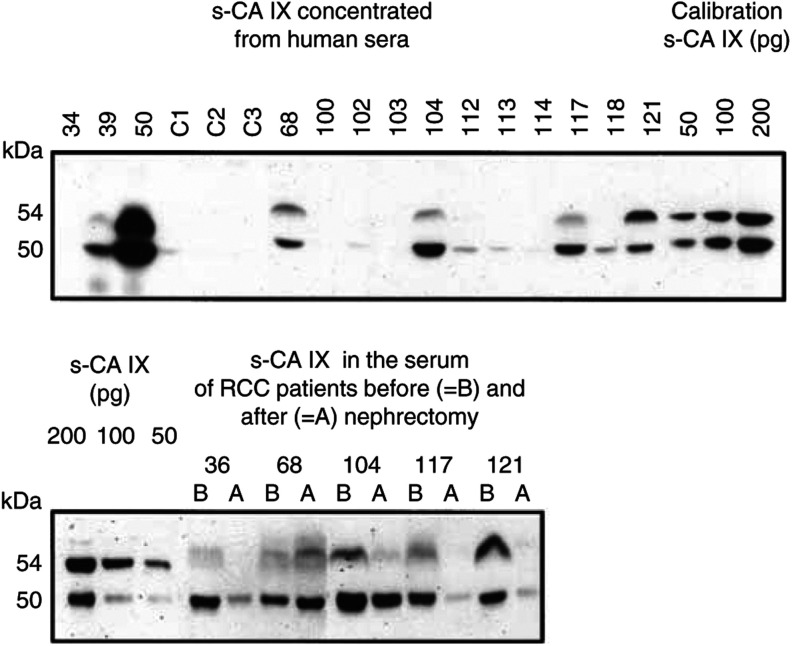
Carbonic anhydrase IX protein in the sera of RCC patients and of control individuals. Each lane was loaded with immunoprecipitate corresponding to original 0.6 ml of the serum. Lane designation: C1–C3=healthy blood donors; 100=stones in the urether; non-RCC tumours: 39=papillary carcinoma from renal cells, 103=urothelial tumour of renal pelvis, 121=sarcomatoid renal carcinoma; all other samples=RCC (the largest are no. 68=2350 cm^3^, no. 36=1466 cm^3^, no. 50=725 cm^3^, and the smallest no. 102=9 cm^3^, no. 112=14 cm^3^).

An example of our tests is shown in Figure 3

(top panel). There does not seem to be a clear correlation between the tumour size and the concentration of CA IX in the serum. All sera evaluated as ‘negative’, including sera of healthy blood donors, did in fact show an extremely weak 50 kDa band of s-CA IX, which became well visible only after prolonged exposure of the blots.

To obtain more support for the presumed origin of blood s-CA IX from the tumours, we tested several paired sera from RCC patients before and after nephrectomy (Figure 3, bottom panel). Indeed, in three cases the postoperation sera contained much less of s-CA IX than the corresponding preoperation sera. One exception was patient no. 68, showing a postoperation increase of the s-CA IX level – but this patient underwent only a palliative operation. Only a moderate decrease of s-CA IX was seen in patient no. 104; in his case sample A was taken only 1 day after nephrectomy, whereas in all other patients it was obtained 5 days after operation.

### CA IX in urine

**Figure 4:**
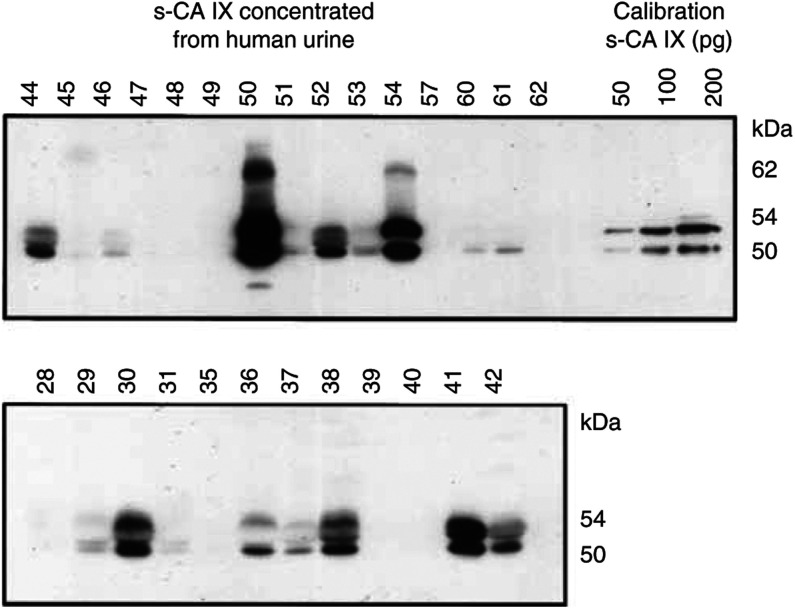
Carbonic anhydrase IX protein in the urine of RCC patients and control individuals. Each lane was loaded with immunoprecipitate corresponding to original 8 ml of urine. Nontumour patients: nos. 45 and 48=stones in the urether; non-RCC tumour patients no. 28 and 57=angiolipoma and no. 39=papillary carcinoma. All other samples=RCC (the largest were no. 36=1466 cm^3^, no. 50=725 cm^3^, the smallest no. 44=1 cm^3^, no. 60=12 cm^3^ and no. 38=14 cm^3^).

The RCC patients excrete s-CA IX protein in urine (Figure 4

). For obvious reasons, we could afford concentrating the antigen from 10 ml of urine (or from larger volumes if needed), and loading one lane on the gel with an aliquot corresponding to 8 ml. It is turning out that a relatively high percentage (19/27=70%) of the patients excrete CA IX in urine. *M*_r_ of the protein is about the same as in blood. Some of the urine samples showed additional bands of intermediate size. In two strongly positive samples (nos. 50 and 54), there was an additional band of 62 kDa. Control specimens of urine from 25 healthy persons, two nontumour and three non-RCC tumour urology patients were all CA IX negative.

The data on CA IX concentration in biological materials tested in the course of these experiments are summarised in Table 1

**Table 1:** Concentration of CA IX in biological materials

HT29 cell extract^a^	4 mg g^−1^ total protein
Fresh RCC extract	0.5–2 mg g^−1^ total protein
Normal kidney extract	Not detectable
HT29 TC medium^a^	20–50 ng ml^−1^
RCC TC media	20–50 ng ml^−1^
Sera of RCC patients	20 pg–3.6 ng ml^−1^
Control sera	5–25 pg ml^−1^
Urine of RCC patients	20 pg–3 ng ml^−1^
Control urine	0–2 pg ml^−1^

a A 5-cm Petri dish corresponds to 5 ml of medium or to 0.5 mg total protein extracted from the cell monolayer.

. The figures for cell and tumour extracts and for TC media are based on ELISA. The CA IX concentration in blood sera and in urine was determined by densitometric reading of films exposed with Western blots and calculated by a comparison with included calibration with HT29 medium, titrated in ELISA. All these figures must be taken only as very approximate, due to high biological variability (density-dependent expression of CA IX in cell cultures, volume of fluids consumed by the patients before providing the urine for CA IX determination). Western blotting is only a semiquantitative method and no clearcut borderline exists between ‘normal’ and ‘elevated’ concentrations of the antigen.

### Recovery of CA IX 50 and 58 kDa bands from the diluted cell extract or TC medium

In the course of these experiments, we made an unexpected observation, which is relevant mainly from the methodical point of view, nevertheless, it cannot be omitted from this paper, since it was also encountered but not explained in other laboratories.

The concentration of CA IX in the serum and urine of RCC patients is too low to be detected without previous concentration. Therefore, we tested how efficiently CA IX can be immunoprecipitated from diluted cell extracts or TC media of HT29 cells.

**Figure 5:**
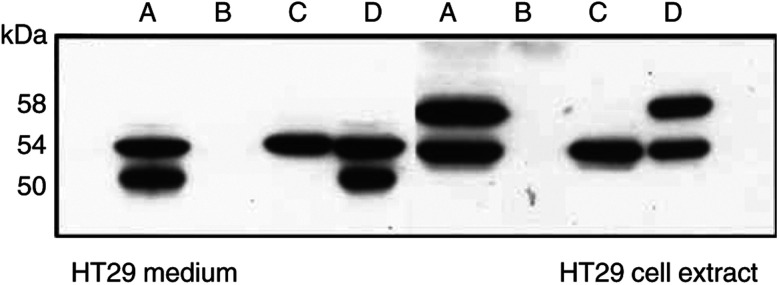
Recovery of CA IX bands 50 and 58 kDa from diluted fluids after immunoprecipitation. A=original TC medium or extract from HT29 cells. To the same antigens as in A was added mAb M75 (ascites fluid) and Protein A-Sepharose and after appropriate incubation, the mixtures were centrifuged and analysed; B=supernatant; C=pellet. With antigens from the medium, B+C was mixed and analysed=D; with antigen from cell extract, to the pellet was added 2% FCS (=D). All samples for the analysis were adjusted to the same concentration of CA IX protein.

The result was somewhat puzzling (Figure 5

). The original cell extract or TC medium contained two bands of CA IX. However, after addition of M75 antibody followed by PA-Sepharose beads and low-speed centrifugation, no CA IX was detected in the supernatant and all CA IX antigens were present in the pellet. Surprisingly, it was seen only as a single band of 54 kDa, no matter whether it was from the cell extract or from the medium. The other band had disappeared. When the pellets were mixed with corresponding supernatants, the lost bands appeared again in their original intensity. For re-appearance of the lost band, it was sufficient to add FCS to a final concentration of 1–2%. This observation suggests that FCS (or various other proteins) provides some sort of steric support for CA IX on Western blots. In this experiment, the p54 band did not disappear after adding the mAb and Protein A–Sepharose and after centrifugation, most likely because during migration in polyacrylamide gel electrophoresis and blotting it overlaps with the denatured heavy chain of M75 IgG. Since this observation, we started to add 1.5–2% of FCS to Laemmli sample buffer to extract CA IX from the immunoprecipitates and to dilute the samples before gel electrophoresis and Western blotting.

## DISCUSSION

CA IX is the only member of the family of CAs that shows a strong association with several types of human cancer. Normally, it is present only in mucosa of the alimentary tract, mainly in the stomach, but on the other hand, its expression in the tumours is ectopic as a rule. It is a relatively complex molecule, exerting at least two functions. Both of them may be of importance in normal alimentary tract as well as in tumours. One of them is enzymatic activity; CA IX belongs to carbonic anhydrases with the highest activity (Wingo *et al*, 2001). In addition, CA IX is a cell adhesion molecule, most likely involved in cell-to-cell communication; its binding site overlaps with the M75 epitope (Závada *et al*, 2000).

Recently generated *CA9*−/− mice cast more light on the normal function of CA IX protein: it consists in differentiation and growth regulation of gastric mucosa (Ortová-Gut *et al*, 2002). The role of CA IX in the chain of molecular events in the process of oncogenesis remains to be elucidated. In certain types of tumours, the expression of CA IX reflects hypoxia, resulting in a poor prognosis (for review, see Harris, 2002).

The present paper is focused on the soluble form of the CA IX protein shed by tumour cells. It answers all the questions listed in the introductory paragraph: s-CA IX is being shed from the cells into the culture medium, and compared with cell-associated protein p54/58, it is somewhat shortened (to 50 and 54 kDa). This size corresponds to the extracellular part of the CA IX molecule, which is composed of proteoglycan (PG)-like and CA domains that have been cleaved off the rest of the molecule – the transmembrane (TM) segment and intracellular (IC) tail of CA IX (Opavský *et al*, 1996). The s-CA IX contains both PG and CA domains, since it reacts with both mAb M75 specific for PG and with MAb V-10 specific for the CA domain.

Shedding of CA IX is moderately efficient; 2 days after exchange, the medium contains about 10% of the total amount of CA IX compared with the cell monolayer (Figure 2). On the other hand, the concentration of s-CA IX in the blood of RCC patients, even if they carry very large tumours, is approximately 1000 × lower than in TC media (Table 1). This shows that s-CA IX is rapidly cleared from the blood. There may be several mechanisms of clearance; we have demonstrated one of them – excretion in urine without any major degradation (Figure 4). Concentration of the s-CA IX in the urine of RCC patients corresponds approximately to that in the blood. An apparently higher percentage of CA IX-positive samples of urine from RCC patients (70%), compared with positive samples of serum (40%) has two causes. One of them is a larger starting volume of urine for concentrating CA IX protein, while the other is a relatively higher level of CA IX in normal sera compared with control urine samples.

Possibly, some of CA IX could be degraded to smaller fragments; we are aware of the existence of CA IX-related polypeptides of 20–30 kDa lacking the PG domain. However, their detection and quantification will require other technical improvements. Two urine samples – nos. 50 and 54 – contain an apparently larger protein of 62 kDa reacting with mAb M75. The significance of this larger protein is not clear; it was not found in the blood or in the tumour extract from the same patient.

At present, the quality of Western blots from blood and urine of RCC patients appears to be satisfactory, but admittedly, Western blotting is not really suitable as a diagnostic test for hundreds or thousands of urine samples. The present results merely show that development of appropriate methods is feasible and most likely indeed worthwhile; they may find clinical application for monitoring the patients after surgery and possibly could be helpful in deciding upon the optimum method for therapy.
